# Selenium and selenoproteins in age-related metabolic diseases: mechanistic insights and translational perspectives

**DOI:** 10.3389/fnut.2026.1908597

**Published:** 2026-08-25

**Authors:** Oladayo E. Apalowo, Taiwo S. Adewole, Ismaila F. Yusuf, M. Wes Schilling

**Affiliations:** 1 Department of Biochemistry, Nutrition and Health Promotion, College of Agriculture and Life Sciences, Mississippi State University, Starkville, MS, United States; 2 Department of Biological Science, Florida State University, Tallahassee, FL, United States; 3 Department of Systems Biochemistry, Institute of Biochemistry and Pathobiochemistry, Faculty of Medicine, Ruhr University Bochum, Bochum, Germany

**Keywords:** cardiovascular disease, insulin resistance, MASLD, obesity, selenium, selenoprotein, type 2 diabetes

## Abstract

Selenium is an essential trace element incorporated into selenoproteins, which regulate redox balance, thyroid hormone metabolism, protein folding, and stress signaling. The relationship between selenium and metabolic health is complex, with growing evidence of a U-shaped association. This review synthesizes current evidence on selenium and selenoproteins in age-related metabolic diseases, with a focus on MASLD, obesity, and cardiovascular disease. Mechanistic data from experimental models are integrated with human observational and intervention studies to evaluate how selenium status, chemical form, dose, and timing influence metabolic outcomes. Evidence indicates a narrow biological window for selenium. Selenium deficiency impairs glucose metabolism and antioxidant defenses and promotes liver injury. However, excessive selenium exposure in selenium-replete individuals may exacerbate insulin resistance, adiposity, and cardiometabolic risk. Several selenoproteins, including GPX1, SELENOP, SELENOM, SELENON, SELENOS, SELENOK, DIO2, and SELENOH, are implicated in various metabolic pathways, acting as protective mediators or disease-associated regulators. Their roles depend on tissue type, expression level, and disease stage. Current evidence does not support widespread selenium supplementation but prioritizes dietary sources, restricts supplementation to cases of confirmed or probable deficiency, and emphasizes the U-shaped risk profile associated with selenium exposure.

## 1 Introduction

Demographic aging and declining physical activity have intensified public health concerns about metabolic health (1). Age-related metabolic disorders are characterized by the progressive accumulation of cardiometabolic dysfunction, including obesity, metabolic dysfunction-associated steatotic liver disease (MASLD), and cardiovascular disease (CVD) (2, 3). These disorders are linked by common pathophysiological mechanisms, such as insulin resistance and chronic low-grade inflammation, which render them important targets for research and prevention efforts addressing age-related morbidity (2).

Empirical evidence indicates that glucose tolerance declines with age across species, a process mediated by increased adiposity, particularly visceral fat (4). These physiological alterations disrupt lipid metabolism and glucose regulation, mechanisms that underlie the elevated risk of age-related diseases and reduced longevity observed in older adults (5, 6). The nutrient-sensing pathway is a conserved system that regulates cellular metabolism and stress responses. It involves signaling cascades (PI3K/Akt, Ras/MEK/ERK), hormones (insulin, IGF), and key regulators such as MTORC1 that respond to nutrients and stress (7). This network controls critical processes, including autophagy, protein synthesis, and metabolism. When nutrients are abundant, they promote growth (anabolism); when scarce, they activate stress defense mechanisms. Reducing this pathway's activity has been shown to extend lifespan and healthspan in animal models (e.g., *Caenorhabditis elegans, Drosophila melanogaster*, and mouse models) (8, 9).

Selenium (Se) is an essential trace element in many organisms and is incorporated as selenocysteine (Sec) during translation at the active sites of twenty-five selenoproteins in humans and twenty-four in rodents. The primary biological functions of Se are mediated through selenoproteins, which are defined by the presence of one or more Sec residues (10). The relationship between Se and the incidence of metabolic disorders remains unclear. Both human and animal studies have reported positive, negative, or neutral correlations between serum Se and glucose levels (11). Evidence indicates a U-shaped association between Se status and disease risk, suggesting that both insufficient and excessive intakes may contribute to the development of chronic metabolic diseases (12, 13).

Se deficiency may affect immune system performance, thyroid hormone metabolism, antioxidant defense systems, endocrine and neurological issues, cardiovascular health, and Keshan and Kashin-Beck diseases (14). On the other hand, excessive Se intake can be detrimental to human health. The Tolerable Upper Intake Level for Se is 400 μg/day for all people, including those who are pregnant or nursing. At the same time, the Recommended Dietary Allowance for adult men and women aged 19 and older is 55 μg/day (15, 16).

In nature, Se mainly exists in two chemical forms: organic and inorganic. Inorganic Se exists in several forms, including selenate (${\text{SeO}}_{4}^{2-}$), selenite (${\text{SeO}}_{3}^{2-}$), selenide (Se^−^), and elemental Se (Se^0^) (17). Among these, selenate (${\text{SeO}}_{4}^{2-}$) is the most common inorganic form found in nature. Organic Se is mostly found in selenoamino acids like selenomethionine (SeMet) and Sec, where Se replaces sulfur (S) in the structures of methionine (Met) and cysteine (Cys) (18). Because Se concentrations in soil vary geographically, endogenous Se levels in plants differ across locations and among populations (19).

Although Se and selenoproteins are implicated in the regulation of oxidative stress, inflammation, mitochondrial integrity, and lipid metabolism, their specific roles in chronic metabolic and cardiovascular states remain unclear. Previously referred to as non-alcoholic fatty liver disease (NAFLD), MASLD is a widespread chronic liver condition characterized by excessive TG accumulation that affects over 30% of the global population and arises from complex interactions among cardiometabolic and environmental risk factors (20, 21). Evidence indicates that insulin resistance is central to the development of metabolic and cardiovascular disorders. Clinically, interventions targeting insulin resistance and endothelial dysfunction have been shown to lower cardiovascular risk (22).

Moreover, existing evidence from experimental and clinical studies is fragmented, with inconsistent findings regarding the effects of Se deficiency, supplementation, adequacy, or excess on metabolic disease. Metabolomic analyses of Se-deficient livers remain inconclusive, as some studies report changes in lipid synthesis. Meanwhile, others observe increased lipid peroxidation and modifications in acylcarnitine and phosphatidylcholine profiles (23). Recently, Liu et al. (24) and Xu et al. (16) summarized the mechanisms of Se supplementation and the risks of supranutritional Se intake in MASLD. However, a comprehensive review is warranted to synthesize current knowledge on Se supplementation and deficiency, elucidate mechanistic pathways, and assess the potential of selenoproteins as biomarkers and therapeutic targets in MASLD, obesity, and CVD.

Hence, this narrative review: (1) examines the influence of Se status on hepatic oxidative stress, inflammation, mitochondrial dysfunction, endoplasmic reticulum stress, and lipid metabolism, all of which contribute to chronic metabolic risks; (2) evaluates experimental and clinical evidence linking Se intake, circulating Se biomarkers, and specific selenoproteins to MASLD, obesity, and CVD; and (3) identifies existing knowledge gaps and outlines future research directions for Se-related precision nutrition and biomarker development for age-related metabolic diseases.

## 2 Se intake and metabolism

Dietary Se exists primarily as selenomethionine (SeMet), Sec, selenite (${\text{SeO}}_{3}^{2-}$), and selenate (${\text{SeO}}_{4}^{2-}$), all of which exhibit high bioavailability and are readily absorbed (25). Additionally, SeMet, the predominant dietary form, serves as an unregulated Se pool that provides a reserve for the body's Se needs (25). Major dietary sources include organ meats and seafood, followed by grains, cereals, and dairy products; environmental sources include cigarette smoke, air, soil, and water (26). Optimal Se intake depends on multiple factors: mechanism of action, Se species, pathological condition, nutritional status, genetic variability, and lifestyle (27). Daily Se intake and plasma/serum concentrations differ across countries (28). The US recommended dietary allowance (RDA) for adults is 55 μg/day (29) and WHO recommendations (26–34 μg/day) (30). However, the average U.S. intake (93–151 μg/day) exceeds both the US RDA and WHO recommendation. The high Se intake in the US is due to high soil levels, which are reflected in food, and to more people taking supplements. About half of the US population takes dietary supplements, and around 18% to 19% of adults and children use supplements that contain Se (199).

The liver, the first organ to encounter absorbed Se, accumulates significant amounts and serves as the primary site for SeMet entry into the Se pool via its active transsulfuration pathway (25). Hepatic selenide follows two pathways: selenoprotein synthesis or conversion to excretory metabolites. Two selenoproteins directly regulate Se metabolism: SEPHS2 converts selenide to monoselenophosphate during Sec synthesis, while SELENOP transports Se to peripheral tissues. Monoselenophosphate enables the conversion of phosphoseryl-tRNA[ser]sec to selenocysteyl-tRNA[ser]sec, the Sec donor for selenoprotein translation (25). Intracellular Se recycling sustains selenoprotein synthesis even under Se-deficient conditions (31).

## 3 Selenoprotein functions

**Figure 1:**
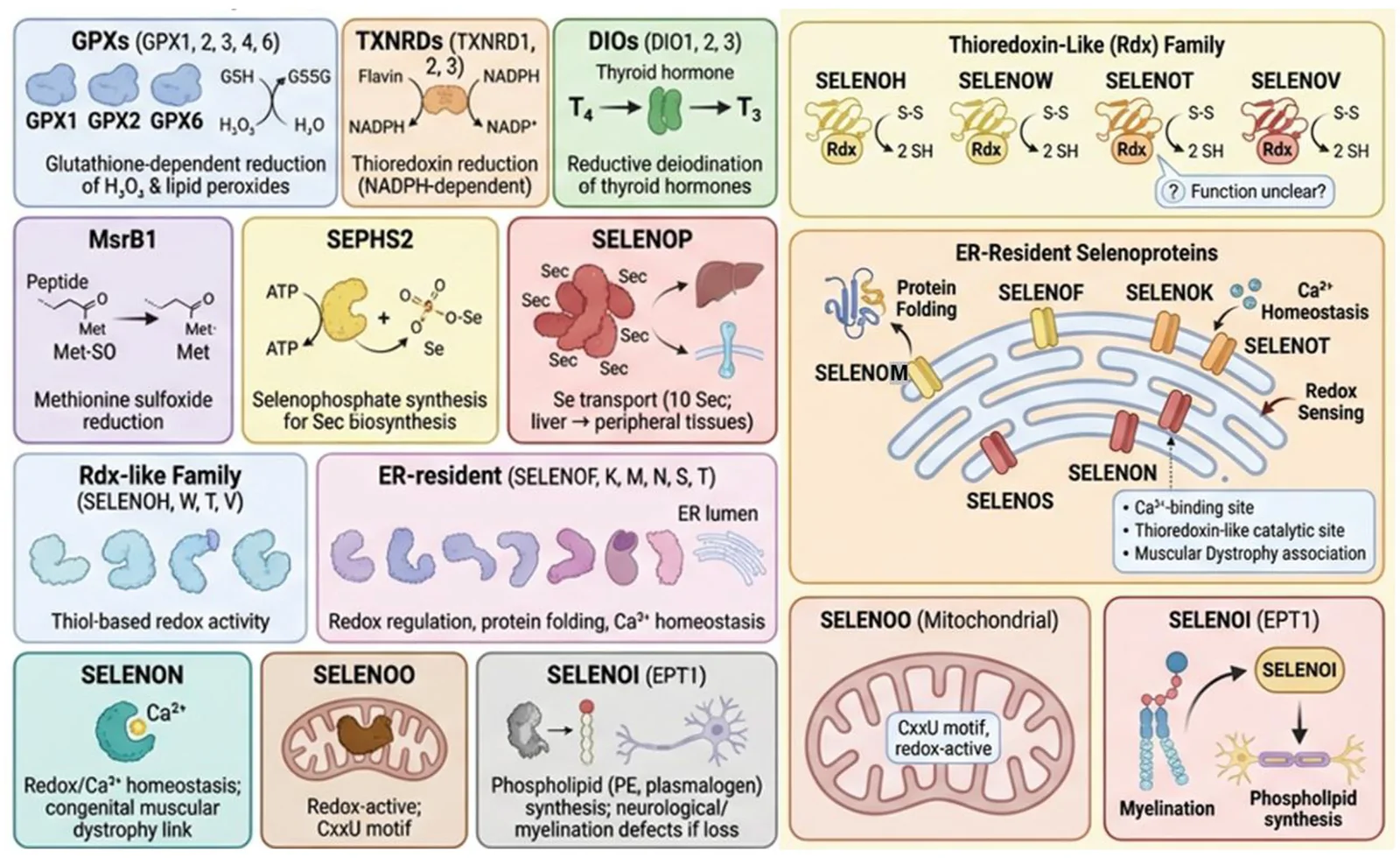
Human Selenoproteins: Families, functions, and cellular localizations.

Almost all selenoproteins are oxidoreductases with Sec as the catalytic residue (32). Due to its strong nucleophilicity and low reduction potential, Sec reacts rapidly with peroxides under physiological conditions. This reactivity facilitates redox cycling, enabling selenoproteins to modulate transient oxidative signals that are essential for insulin receptor tyrosine kinase activation and subsequent PI3K/Akt signaling (33). In humans, approximately five GPX (GPX 1, 2, 3, 4, and 6) selenoproteins catalyze the glutathione (GSH)-dependent reduction of hydrogen peroxide and phospholipid peroxides (Figure 1) (32, 34). Sec selenolate directly attacks peroxides, forming a transient selenic acid (R-SeOH) intermediate that GSH rapidly reduces. The absence of a regenerating pocket and the surface accessibility of Sec contribute to exceptionally rapid kinetics, which enable GPxs to efficiently scavenge H_2_O_2_ generated during insulin receptor activation (18). SEPHS2 is essential for Sec biosynthesis because it catalyzes the ATP-dependent formation of selenophosphate, the Se donor for Sec (35).

Thioredoxin reductases (TXNRD1, TXNRD2, and TXNRD3) contain Sec near the C-terminus and utilize NADPH to reduce oxidized thioredoxin. The iodothyronine deiodinase (DIO1, DIO2, and DIO3) family of selenoproteins, which possess Sec within an N-terminal canonical thioredoxin (Trx) fold, facilitates the reductive deiodination of thyroid hormones. This structural organization allows functional specialization in redox metabolism and signal transduction (18, 36). Methionine-R-sulfoxide reductase (MsrB1) is involved in the reduction of oxidized methionine residues in proteins (37). The remaining twelve selenoproteins either lack a clearly defined function or possess only partially established roles (34). Among these are the thioredoxin-like (Rdx) family of selenoproteins (SELENOH, SELENOW, SELENOT, and SELENOV), which exhibit thiol-based redox activity (38).

SELENOP transports Se to peripheral tissues and binds several metals associated with neurodegenerative diseases, including Fe^2+^, Al^3+^, Cu^2+^, and Zn^2+^, via its His-rich domain (39). It contains ten Sec residues, one located in the large N-terminal region and nine in the smaller C-terminal region, and is predominantly synthesized in the liver (40–42). The presence of multiple Sec residues increases the density of redox-active sites, allowing SELENOP to function as both an antioxidant and a Se reservoir (43). This modular structure enables interaction with receptors such as ApoER2, potentially linking circulating SELENOP levels to β-cell function, systemic redox signaling, and insulin sensitivity.

Endoplasmic Reticulum (ER)-resident selenoproteins, such as SELENOF, SELENOK, SELENOM, SELENON, SELENOS, and SELENOT, perform diverse functions. This includes redox sensing and regulation, protein folding, and calcium homeostasis (44). SELENON contains a calcium-binding site, TXNRD-like catalytic sites, and is associated with congenital muscular dystrophy, leading to spinal rigidity and restrictive respiratory syndrome (45). SELENOO is a mitochondrial CxxU-containing redox-active protein expressed in multiple tissues (46). SELENOI, also known as ethanolamine phosphotransferase 1 (EPT1), is involved in phosphatidylethanolamine and plasmalogen synthesis, and loss-of-function mutations linked to several neurological and myelination defects (47).

The human selenoproteome is a diverse network of redox-active enzymes and structural proteins, unified by the incorporation of Sec as a catalytic or structural residue. From GPX and TXNRD, which neutralize reactive oxygen species, to ER-resident proteins involved in proteostasis and calcium homeostasis, selenoproteins occupy critical nodes in cellular redox signaling, metabolic regulation, and stress response. The network's dependence on adequate Se availability underscores the physiological importance of Se status. Dysregulation in selenoprotein expression is associated with metabolic, neurodegenerative, and musculoskeletal diseases. Understanding the structural and mechanistic basis of individual selenoproteins provides a foundation for clarifying how Se affects human health.

## 4 Molecular mechanisms of insulin resistance: pathways and clinical implications

Insulin resistance (IR) is a pathological condition in which insulin-dependent cells, such as skeletal muscle, liver, and adipocytes, the primary targets of intracellular glucose transport and glucose and lipid metabolism, fail to respond appropriately to normal insulin levels in the bloodstream (48, 49). The major metabolic functions of insulin are to increase glycogen synthesis in skeletal muscle, accelerate glucose uptake in adipocytes and skeletal muscle, regulate hepatic glucose metabolism, and inhibit lipolysis in adipocytes (50).

When plasma glucose and amino acid levels rise, pancreatic beta cells secrete insulin to restore these concentrations to physiological levels. Disruption of insulin signaling impairs cellular glucose uptake, leading to hyperglycemia (51). As a characteristic feature of most metabolic diseases, any errors in the expression or activity of several enzymes and modulatory proteins involved in insulin signaling compromise normal metabolic processes and cause IR in peripheral tissues (52). IR and subsequent β-cell dysfunction, both resulting from feedback loops between abnormal insulin production and insulin action, represent two key pathogenic features frequently observed in T2D.

Multiple signaling pathways are critical for modulating the pathological changes associated with metabolic disorders. The PI3K/Akt signaling cascade regulates insulin responsiveness, while the 5′AMP-activated protein kinase (AMPK) pathway inhibits β-cell dysfunction (53). Although the PI3K/Akt pathway is recognized as the primary mediator of insulin action, other classical signaling pathways implicated in T2D include mitogen-activated protein kinase (MAPK), AMPK, WNT, and transforming growth factor beta (TGFβ) pathways (54).

Elevated secretion of tumor necrosis factor-alpha (TNF-α), interleukin-6 (IL-6), monocyte chemoattractant protein-1 (MCP-1), and other products from macrophages and adipose tissue-resident cells contribute to the development of IR (55). Studies in experimental obesity models and obese individuals have demonstrated that increased adipose tissue production elevates TNF-α levels, thereby reducing insulin sensitivity (56). The c-Jun N-terminal kinase (JNK) and IκB kinase-β (IKK-β)/nuclear factor-κB (NF-κB) pathways are activated by TNF-α and IL-6 via receptor-mediated mechanisms, leading to the upregulation of inflammatory mediators that promote IR (57).

Inflammation, ER stress pathway activation, and hepatic lipid accumulation promote IR during lipid dysregulation (58–60). Intracellular diacylglycerols (DAGs) reduce insulin signaling by activating novel protein kinase C (PKC) isoforms that inhibit insulin receptor kinase-mediated phosphorylation of insulin receptor substrates 1 and 2. In contrast, intracellular ceramides inhibit Akt2 activation (61). Adipocytokines such as TNF-α, interleukin-1β (IL-1β), and IL-6 impair insulin signaling by activating the JNK or IκB kinase-β pathways (62). The unfolded protein response and ER stress pathways also contribute to IR (63). Key mediators of ER stress, including dsRNA-activated protein kinase-like ER kinase (PERK), activating transcription factor 6 (ATF6), and inositol-requiring enzyme 1 (IRE1), dissociate from immunoglobulin heavy-chain binding protein (BiP), thereby initiating a response that impairs insulin signaling through JNK activation (64).

IR and endothelial dysfunction are early indicators of CVD (22). Endothelial cells facilitate the transport and distribution of nutrients and hormones. This includes glucose, fatty acids, and insulin. They are involved in the elimination of metabolic waste products within resistance vessels, thereby regulating blood flow and systemic blood pressure (65). Manifestations of IR in the vascular system include impaired vasodilation, retinopathy, nephropathy, increased vascular inflammation, and atherosclerotic lesion formation (66).

Evidence suggests that endothelial nitric oxide (NO) production is essential for vasodilation and that insulin-induced activation of the PI3K pathway contributes to basal NO release (67). Activation of PI3K in vascular endothelial cells is necessary to enhance endothelial NO synthase (eNOS) production and activity in response to insulin. Defects in the PI3K-dependent pathway, which is necessary for insulin-stimulated eNOS production and activity in vascular endothelial cells, could explain the frequent association between vascular disease and IR across multiple tissues.

The redox activity of selenoproteins is positioned at a key regulatory node within the insulin signaling pathway, as insulin signaling is inherently redox-sensitive. Insulin binding to its receptor activates the PI3K/AKT signaling pathway, which mediates essential cellular responses (68). Redox mechanisms regulate this pathway. Typically, hydrogen peroxide (H_2_O_2_) generated during insulin receptor activation acts as a second messenger, modulating signaling by reversibly oxidizing cysteine residues in phosphatases and kinases (68). Selenoproteins, particularly GPXs and TXNRDs, efficiently regulate these redox signals because Sec is more reactive than cysteine.

### 4.1 Implication of Se in metabolic diseases

**Figure 2:**
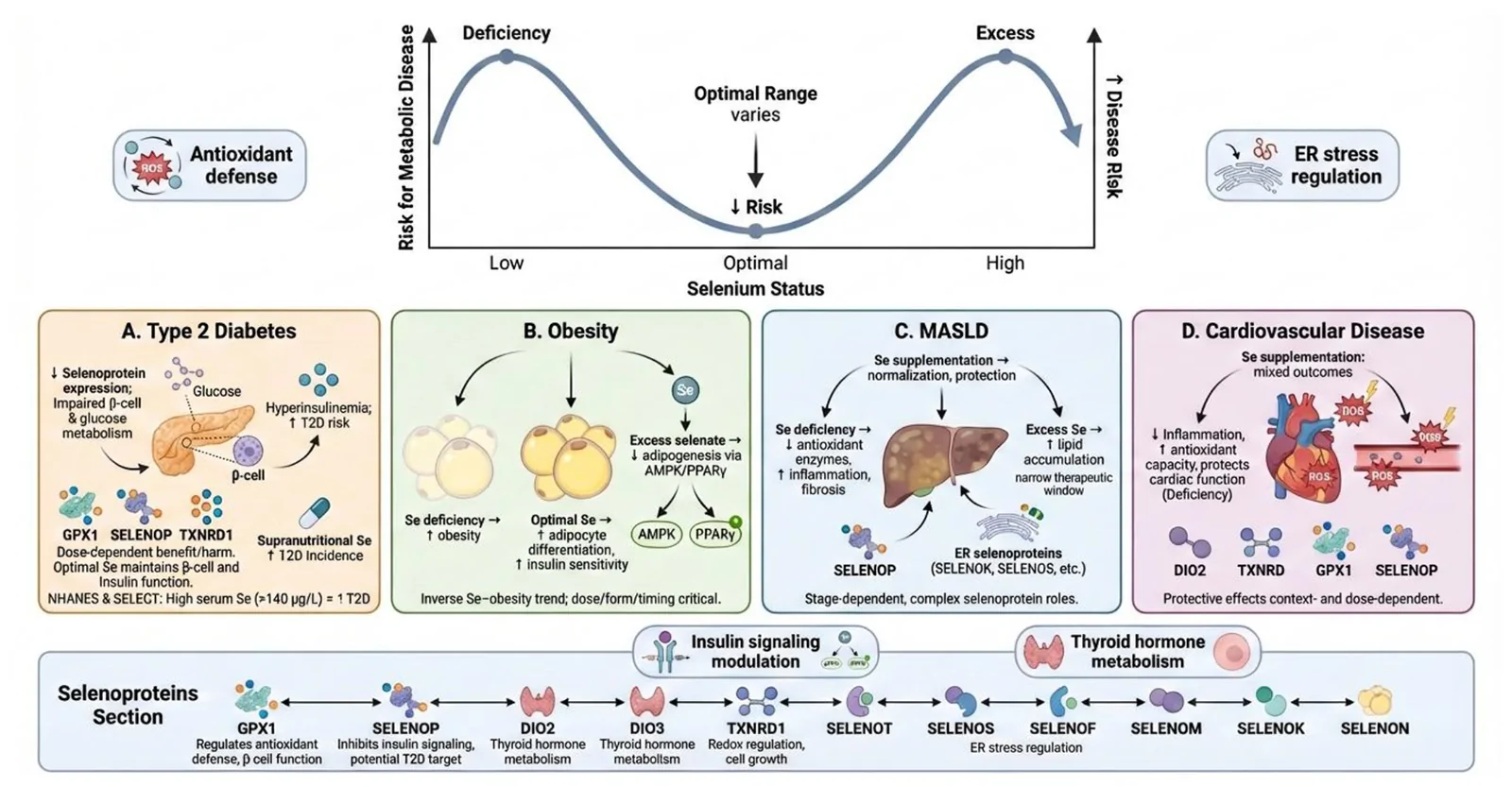
U-shaped effect of Se across major metabolic disease pathways. Both low and high Se levels may result in similar pathological outcomes by acting through distinct, often opposing molecular pathways that ultimately disrupt the same redox-sensitive nodes.

Evidence from mouse and human studies demonstrates the roles of Se and certain selenoproteins in the well-studied T2D (69–76). The relationship between Se and T2D demonstrates a critical U-shaped dose-response pattern, where both deficiency and excess pose significant metabolic risks (Figure 2). Se deficiency impairs glucose metabolism through multiple mechanisms. This includes downregulation of low-hierarchy selenoproteins, compromised pancreatic islet function and insulin production, reduced insulin-stimulated glucose oxidation in adipocytes, telomere shortening and cellular senescence, and diminished antioxidant defense capacity (77–79). These deficiencies manifest clinically as glucose intolerance, IR, hyperglycemia, diabetic complications, albuminuria, and glomerular sclerosis.

Supranutritional Se intake, particularly in Se-adequate populations, is associated with increased T2D risk, hyperinsulinemia, elevated fasting glucose, and worsened IR, as evidenced by large epidemiological studies (National Health and Nutrition Examination Survey (NHANES), Se and Vitamin E Cancer Prevention Trial (SELECT)), and animal models (80–82). The SELECT trial provides the most comprehensive randomized controlled trial (RCT) evidence regarding Se supplementation and diabetes risk. This four-arm study included 35,533 male participants who received 200 μg/day of Se as L-SeMet. The findings indicate that supplementation with 200 μg/day of Se results in a non-significant increase in the risk of T2D when Se is administered alone (RR = 1.07, 95% CI: 0.94–1.22, P = 0.16) (81). While severe Se deficiency is detrimental, supranutritional Se intake, whether from supplements or elevated baseline levels, is associated with a higher incidence of T2D.

In specific contexts such as streptozotocin-induced diabetes, Se supplementation (Na_2_SeO_3_) demonstrates therapeutic benefits by reducing oxidative stress, inflammation, and glycemic markers, suggesting that high Se status may modulate the transcriptional regulation of proinflammatory cytokines in chronic, low-grade inflammation associated with obesity-related IR and T2D (83–85). These paradoxical findings underscore that Se's effects on glucose metabolism are influenced by baseline Se status, dose, chemical form, duration of exposure, and underlying metabolic state.

The multifaceted roles of selenoproteins in T2D pathogenesis reveal a complex regulatory network where expression levels determine whether these proteins protect against or contribute to metabolic dysfunction. While GPX1, SELENOP, and several members of the thioredoxin-like family contribute to glucose homeostasis and insulin signaling, their paradoxical effects underscore the challenges of therapeutic intervention (86). Hypertension frequently co-occurs with diabetes and obesity, driven by a proinflammatory state marked by an increase in systemic adrenergic activity, dyslipidemia, and hyperglycemia. Given Se's narrow therapeutic window and the tissue-specific requirements for selenoproteins, effective treatments must move beyond systemic approaches toward targeted modulation of individual selenoproteins in specific metabolic tissues.

## 5 Se and MASLD

Previous research indicates that Se plays a significant role in hepatic lipid metabolism (16, 23, 87–90); however, the precise function remains uncertain. Se deficiency has been associated with both decreased hepatic lipid levels and inconsistent alterations in specific lipid metabolites (87, 91), increased oxidative stress, and accelerated progression of hepatic fibrosis (92). While Se supplementation has been found to improve liver function (93, 94), some studies have shown that higher Se intake may accelerate MASLD progression (95, 96). Se may offer therapeutic potential in MASLD research and treatment by targeting key disease mechanisms, including oxidative stress, inflammation, insulin resistance, and lipid dysregulation, which have shown limited response to agents such as vitamin E. Unlike generic antioxidants or other essential trace elements that act mainly as cofactors, Se is co-translationally incorporated as Sec into selenoproteins, helping to maintain liver redox balance.

### 5.1 *Se* deficiency and MASLD

Se deficiency has been shown to reduce the expression of intracellular antioxidant enzymes, such as SOD1, contributing to the development of fatty liver (97). Se deficiency may promote inflammation through a multi-hit mechanism by activating the NF-κB pathway at the organ level (16). In a recent study using a pure-line pig model of Se deficiency-induced hepatic pathogenesis, diets containing either 0.07 mg/kg Se or 0.3 mg/kg Se (supplemented with SeMet) for 16 weeks led to oxidative imbalance, as evidenced by altered selenoprotein expression at both the mRNA and protein levels (87). This intervention also impaired the glutathione antioxidant system, leading to increased glutathione synthesis and catabolism, indicating that the liver is metabolically susceptible to oxidative imbalance caused by Se deficiency.

A rat model of hepatic fibrosis induced by N-nitrosodimethylamine (NDMA) demonstrated that reduced Se and GPx levels impaired cellular antioxidant defenses, leading to increased oxidative stress and accelerated progression of hepatic fibrosis (92). In patients with liver cirrhosis, Se concentrations were found to be lower than normal plasma levels and declined in proportion to disease severity (98). Previous studies also showed that Se deficiency in animals resulted in liver necrosis, and significantly reduced Se content was detected in the liver of patients who died from cirrhosis compared to healthy controls (99). Hence, Se appears to play a protective role in maintaining liver integrity by supporting antioxidant capacity. Its depletion serves as both a marker and a potential contributor to the severity of hepatic damage, including fibrosis and cirrhosis.

### 5.2 Se supplementation and MASLD

Se supplementation has been shown to normalize liver function tests, reduce stellate cell numbers and fibrosis following CCl4-induced liver injury, and improve liver function by decreasing malondialdehyde levels and inflammation while enhancing GPx activity (93, 94). Furthermore, two *in vivo* animal studies on MASLD progression demonstrated that co-supplementation with zinc and Se, using Na_2_SeO_3_ at 1.1 mg Se/kg, which exceeds the recommended Se intake for rats of approximately 0.2 mg/kg, could delay MASLD progression (100, 101). Among the various forms shown to reduce oxidative stress and inflammation, which are two primary factors contributing to MASLD progression, organic Se and Se nanoparticles demonstrate superior safety profiles and bioavailability compared to common inorganic forms (24). However, other *in vivo* animal studies and human research have reported that higher Se intake is associated with increased hepatic lipid accumulation (95, 96, 102), suggesting that higher Se intake may accelerate MASLD progression.

Recent human research has indicated an unexpected association between elevated body Se levels and adverse blood profiles of glucose, HbA1c, and lipids (LDL-c, HDL-c, TGs, ApoB, and ApoA1), as well as increased susceptibility to diabetes, even in Se-replete populations such as those in the United States (80, 82, 103–105). In such populations with adequate Se intake, additional intake from supplements or diet can elevate Se levels beyond physiologic needs. At these higher levels, benefits do not increase, and adverse effects become more likely; elevated blood Se levels correlate with poorer glycemic markers. This is most likely explained by a U-shaped relationship, in which at supranutritional levels Se can shift metabolism in an unfavorable direction, compromising metabolic function. Although these studies raise concerns regarding the potential adverse cardiometabolic effects of elevated Se levels, further mechanistic research is required to establish causal relationships and elucidate the underlying molecular mechanisms.

As shown in Tables 1, 2, Se supplementation at appropriate doses demonstrates protective effects, such as normalization of liver function, reduction of fibrosis and inflammation, and enhancement of antioxidant capacity. In contrast, excessive Se intake promotes hepatic lipid accumulation and may accelerate MASLD progression, which underscores the narrow therapeutic window of Se. This observation supports the use of Se as a potential adjunctive strategy for MASLD. This inference further emphasizes the importance of a narrow, safe range and the need for further human intervention trials before definitive clinical recommendations can be established.

**Table 1:** Summary of experimental studies (animal model) on the association of Se with MASLD progression.

References	Experimental animal model	Se treatment	Histopathological, molecular, and biochemical assessment^‡^	Key findings
Han et al. (180)	Sprague-Dawley rats; male/female; 18-day-old	Se-deficient diet fed only AIN-93M formula without sodium selenate (Na_2_SeO_4_); Rats were fed for 109 days.	Histopathology examination Hepatic periportal fibrosis Thickened vessel walls Collagen fiber hyperplasia Bile duct hyperplasia Molecular analysis ↑ MMP1 (1.2–1.8-fold)^†^ ↑ MMP3 (1.5–1.9-fold)^†^ ↓ TIMP1 (0.53-fold) ↓ TIMP3 (0.82-fold) ↑ Matrix degradation activity	A low-Se diet led to Se liver depletion and fibrosis
Qiao et al. (181)	Sprague-Dawley rats; male; raised for 12 weeks	Low-Se diet (0.02 mg Se/kg); Nano-Se supplement-1 (0.10 mg Se/kg); Nano-Se supplement-2 (0.20 mg Se/kg).	Histopathology Disorganized hepatic lobules Irregular hepatocytes Endothelial cell damage Inflammatory cell clustering Portal fibrosis Abnormal hyperplasia Atrophied mitochondria Mitophagy changes Blurred cristae Immunohistochemistry ↓ mTOR and p-mTOR expression ↑ ULK1 and p-ULK1 expression ↓ Staining in portal and central venous regions	Nano-Se supplements may slow the progression of liver fibrosis caused by Se deficiency
Wang et al. (182)	C57BL/6 mice; male; 8-week-old	Se-enriched spirulina (Se content 0.45 mg/kg); Mice were fed for 12 weeks.	Histopathology ↓ Lipid accumulation ↓ Liver fibrosis ↓ Steatosis ↑ Hepatic function Biochemical markers ↓ ALT (36%) ↓ AST (29%) ↓ TC (44%) ↓ TG (22%)	Se supplementation improved hepatic damage recovery, IR, and reduced lipogenic gene expression
Zhang et al. (89)	C57BL/6 mice; male; 6-week-old	Low dose organic Se group L-SeC group (1.0 mg/kg L-selenocysteine); L-SeM group (0.6 mg/kg L-selenomethionine); L-MeSeC group (0.6 mg/kg L-selenomethylcysteine); High dose organic Se group H- SeC group (2.0 mg/kg L-selenocysteine); H-SeM group (1.2 mg/kg L-selenomethionine); H-MeSeC group (1.2 mg/kg L-selenomethylcysteine); Mice were fed for 16 weeks.	Histopathology^±^ ↓ Steatosis score (29–32%) ↓ Fibrosis score (27%) Biochemical markers^±^ ↑ SELENOP (4–21%) ↑ GSH-Px activity (11–36%) ↓ ALT and AST (9–28%) ↓ TG (17–40%) ↓ TC 12% ↓ LDL-C 13% ↓ Hepatic 5-HT (30–38%) Restored hepatic function	Organic Se reduced fat weight and improved hepatic steatosis and fibrosis
Ding et al. (93)	C57BL/6J mice; male; 4–6 weeks old	Se supplementation (anhydrous sodium selenite 4 mg/L); Mice were fed for 4 weeks.	Histopathology ↓ Inflammation ↓ Hepatic fibrosis ↓ Stellate cells No change in necrosis caused by CCl4 Biochemical markers ↑ Liver Se (20%) ↑ Serum ALT (15%) Molecular analyses ↑Apoptotic hepatocytes ↑ Fas (1.42-fold) ↑ MMP-9 (1.46-fold) ↑ TIMP-2 (1.18-fold) ↓ Caspase 8 (0.67-fold) ↑ BAX (1.23-fold) ↑ Bcl Xs/l (1.23-fold)	Se supplementation reduced hepatic inflammation and fibrosis but increased apoptosis
Zhang et al. (183)	C57BL/6 mice; male; 6 weeks old	Se-enriched green tea group (Se = 2.92 ± 0.29 mg/kg); Mice were fed for 4 weeks.	Histopathology Hepatic fibrosis alleviation ↓ Collagen type I (50%) ↓ Collagen type III (75%) ↓α-smooth muscle actin (10%) Biochemical markers ↓ AST/ALT ratio (8%) ↓ Hepatic HA (19%) ↓ Hydroxyproline (49%) ↑ Liver GPx (44%) ↑ SELENOP (62%)	Se-enriched green tea ameliorated hepatic fibrosis and inhibited the activation of hepatic stellate cells by intestinal 5-hydroxytryptamine-hepatic 5-hydroxytryptamine receptor signaling
Liu et al. (184)	Wistar rats; male	Se-enriched probiotics, SP (0.3 μg/g); Rats were fed for 7 weeks	Histopathology ↓ Liver inflammation and necrosis Biochemical markers ↓ ALT (48 %) ↓ AST (27%) ↓ Hydroxyproline (42%) Molecular analyses ↓α-SMA (0.6-fold) ↓ Procollagen-I (0.5-fold) ↓ TGF-β1 (0.4-fold) ↓ TIMP-1 (0.4-fold)	Se-enriched probiotics attenuated CCl_4_-induced hepatic damage, inflammation, and fibrosis, and induced apoptosis of activated hepatic stellate cells
Miyata et al. (185)	Fxr-null mice; male; 4 months old	Selenoneine (0.3 mg Se/kg); Mice were fed for 4 months.	Histopathology No significant alterations in hepatic morphology Hepatic steatosis amelioration Biochemical markers ↓ Hepatic total Se (32%) ↓ Blood clot concentration (81) ↓ Serum ALT (50%) ↓ Serum ALP (54%) ↓ Hepatic AST (40%) ↓ Bilirubin (36%) ↓ Hepatic TG (50%)	Selenoneine reduces hepatocellular damage and liver steatosis in a mouse model of MASLD
Ozardali et al. (94)	Wistar rats; male	Na_2_SeO_4_, 0.2 mg/kg b.w.t; Administered three times a week for a period of 7 weeks.	Histopathology ↓ Necrotic, fibrotic, and cirrhotic processes Restored regular liver architecture Biochemical markers ↓ AST (5%) ↓ ALT (4%) ↓ ALP (54%) ↓ GGT (26%)	Se supplementation lessens CCl4-induced hepatocellular damage characterized by liver necrosis, fibrosis, and cirrhosis
Qiao et al. (186)	Sprague-Dawley rats; male; 21 days old	Se-deficient diet (containing 0.02 mg Se/kg); Rats were fed for 4 weeks	Histopathology Poorly defined hepatocytes Irregular nuclear morphology Vacuolated lipid droplets Abnormal mitochondria Blurred portal vascular structure Mild hyperplasia Thinned, discontinuous central vein endothelium More obvious portal fibrosis Interconnecting fibrosis Slight central venous enlargement Biochemical markers ↓ Serum Se (2-fold) ↓ Liver Se (6-fold) ↓ Akt, p-Akt, mTOR, and p-mTOR	Short-term Se deficiency promotes liver fibrosis by blocking the Akt/mTOR signaling pathway, with a significant contribution from mitochondrial damage
Pant et al. (187)	Sprague-Dawley rats; male; 6–8 weeks	Se-enriched L. acidophilus (L. acidophilus 1 × 10^9^ CFU/ml containing 0.4 μg Se/day); Rats were fed for 12 weeks.	Histopathology ↓ Hepatic vacuolations ↓ Collagen deposition Biochemical markers ↓ TG (67%) ↓ LDL-c (69%) ↓ TC (40%) ↑ HDL-c (80%) ↑ GSH1 (57%) ↓ MDA (55%) Molecular analyses ↑ p-AMPK (7-fold) ↑ SIRT1 (5-fold) ↑ BECLIN1 (2.5-fold) ↑ LC3-II (3-fold)	Se-enriched *L. acidophilus* activates the SIRT-1/AMPK pathway and effectively modulates autophagy and hepatic lipid clearance in MASLD rats
Murano et al. (188)	NSY mice; male; 4 weeks old	Tap water containing 2 mg Se/L SeMet; Mice were kept for 12 weeks	Histopathology Relative amount of hepatic 4HNE unchanged Biochemical markers ↓ Insulin (49%) ↓ Adiponectin (5%) ↓ FFA (16%) ↑ Plasma Se (69%) ↑ Liver Se (103%) Molecular analyses *Liver*: ↑*Gpx1* (5-fold) ↑*Gpx4* (1.5-fold) ↑*Dio1* (10-fold) ↓*Selenop* (0.83-fold) *Muscle*: ↓ Gpx1 (0.9-fold) ↓ Gpx4 (0.7-fold) ↓*Selenop* (0.8-fold) *Pancreas*: ↑*Gpx1* (3-fold) ↑*Gpx4* (2-fold) ↑*Selenop* (2-fold)	HFD caused increased liver fat deposition, SELENOP expression, and IR, while SeMet supplementation increased liver GPx1 expression.
Wang et al. (95)	C57BL/KsJlepr^db^/lepr^db^ diabetic (*db/db*) mice; male; 7 weeks old	Na_2_SeO_4_, 0.8 mg/Kg b.w.t via daily tube feeding; Mice were fed for 9 weeks.	Histopathology Worsened fatty degeneration and vacuolization Numerous swollen hepatocytes Steatosis Hepatic cord congestion. Biochemical markers ↓ Insulin (45%) ↑ Glucose (82%) ↓HOMA-IR (18%) ↓ TG (48%) ↑ TC (32%) ↑ LDL-c (16%) ↓ MDA (27%) ↓ GSH-Px (12%)	Se supplementation reduced hyperglycemia and increased hepatic lipid deposition and inflammatory genes

^‡^For biochemical markers, changes are reported as percent differences relative to control, whereas gene and protein expression data are reported as fold changes (treatment/control).

^±^organic Se (both high and low doses).

^†^Protein and mRNA level.

MMP1, matrix metalloproteinase 1; MMP3, matrix metalloproteinase 3; TIMP1, tissue inhibitor of metalloproteinases 1; TIMP3, tissue inhibitor of metalloproteinases 3; ULK1, unc-51 like autophagy activating kinase 1; HFD, high-fat diet; AST, aspartate aminotransferase; ALT, alanine aminotransferase; CCL4, carbon tetrachloride; TC, total cholesterol; TG, triglycerides; LDL-c, low-density lipoprotein cholesterol; HDL-c, high-density lipoprotein cholesterol; 5-HT, 5-hydroxytryptamine (serotonin); LC-ICP-MS, liquid chromatography–inductively coupled plasma–mass spectrometry; ALP, alkaline phosphatase; AKT, AKT serine/threonine kinase (also known as protein kinase B); MTOR, mechanistic target of rapamycin kinase; 4HNE, 4-hydroxynonenal; DAB, 3,3′-diaminobenzidine; GPX1, glutathione peroxidase 1; GPX4, glutathione peroxidase 4; DIO1, iodothyronine deiodinase 1; GSH, glutathione; AMPK, AMP-activated protein kinase; SIRT1, sirtuin 1.

**Table 2:** Summary of cross-sectional and case-control studies on the association of Se/Selenoprotein with MASLD progression in humans.

References	Observation/ duration	Study participants	MASLD diagnosis	Outcome relative to MASLD^‡^
Choi et al. (189)	Korean Sarcopenic obesity study (KSOS); September 2007–August 2009	20 nondiabetic individuals; Male/Female; MASLD subjects (*n*=60) and control (*n*=60); Age: Control (47.0 ± 13.0), MASLD (49.1 ± 13.1)	Liver attenuation index (LAI) < 5 HU, using unenhanced CT	↑ SELENOP (2.85-fold) SELENOP positively correlated with VFA, hsCRP, baPWV SELENOP negatively correlated with liver attenuation index
Yang et al. (190)	2011 China REACTION study; baseline survey of subsamples from Shanghai in eastern China; 2011–2012	8550 Chinese adults; male/female; ≥ 40 years;	Ultrasonography	↑ Plasma Se (40%) associated with: ↑ TC (4%) ↑ TG (63%) ↑ LDL-c (6%) ↑ HOMA-IR (67%) ↑ ALT (50%) ↑ AST (17%) ↑ GGT (55%) ↓ HDL-c (6%)
Reja et al. (191)	NHANES-III (1988–1994)	A total of 2782 MASLD patients; Male; 20–74 years;	Hepatic ultrasound	A higher serum Se level was significantly associated with a lower likelihood of liver fibrosis.
Shih et al. (192)	NHANES 2017–2018	5641 participants by serum Se quartile: Q1 < 2.21 μg/L Q2: 2.21 to < 2.39 μg/L Q3: 2.39 to < 2.60 μg/L Q4 > 2.60 μg/L); Male/female; 49% men; ≥ 12 years (mean age: 44:88 ± 20.90;	Ultrasound transient elastography	Inverse association between the serum Se and severity of liver fibrosis; Serum Se (>2.60 μg/L): ↑ ALT activity ↓ LSM values
Wang et al. (193)	NHANES: 2011–2012, 2013–2014, and 2015–2016	1319 adults; Male/Female; ≥ 20 years;	Presence of serum ALT >30 IU/L in men and >19 IU/L in women in the absence of other identifiable causes of liver disease	Serum Se: ↑ ALT activity (1.7%) ↑ HOMA-IR (52%) A positive association between serum Se level and MASLD was observed above a serum Se level of 130 μg/L; sex and age did not affect the association.
Wu et al. (102)	Health examinations at Xiangya Hospital, Central South University, Changsha, Hunan, China, October 2013–November 2014	5436 subjects (2587 men and 2849 women); ≥ 40 years; MASLD 1998; Non-MASLD 3438; Dietary Se intake was classified into five categories based on quintile distribution: ≤ 21.3 μg/1000 kcal; 21.4–24.2 μg/1000 kcal; 24.3–26.9 μg/1000 kcal; 27.0–30.5 μg/1000 kcal; ≥ 30.6 μg/1000 kcal	Histological evidence of hepatic steatosis; Absence of specific etiologies of MASLD; Absence of heavy alcohol consumption.	Se intake was positively associated with the OR for MASLD in a dose-response relationship manner (r = 0.88, p = 0.008)
Coelho et al. (194)	Clementino Fraga Filho University Hospital (HUCFF) hepatology clinic; August 2016–December 2017	72 patients; Male/Female; Patients with advanced fibrosis (*n*=32)	Ultrasonography	↓ Serum Se level (6%)
Polyzos et al. (107)	A one-center cross-sectional study carried out between June 2008 and November 2010	31 patients; > 18 years; patients with definite MASH	Ultrasound imaging and abnormal liver tests for at least 6 months	↓ SELENOP (30%)
Chen et al. (88)	First Affiliated Hospital, Medical School, Zhejiang University; 2014	79 patients with MASLD and 79 healthy controls; Age: Non-MASLD 39.5 ± 11.9; MASLD 42.1 ± 10.5;	Hepatic ultrasound	↑ SELENOP (1.17-fold) SELENOP positively correlates with: ↑ ALT (57%) ↑ AST (19%) ↑ GGT (79%) ↑ UA (9%)

^‡^For biochemical markers, changes are reported as percent differences relative to control, whereas gene and protein expression data are reported as fold changes (treatment/control).

NHANES, national health and nutrition examination survey; HSCRP, high-sensitivity C-reactive protein; BMI, body mass index; HOMA-IR, homeostatic model assessment of insulin resistance; GGT, gamma-glutamyl transferase; CRP, C-reactive protein; SCLY, selenocysteine lyase; AST, aspartate aminotransferase; ALT, alanine aminotransferase; TC, total cholesterol; TG, triglycerides; LDL-c, low-density lipoprotein cholesterol; HDL-c, high-density lipoprotein cholesterol.

### 5.3 Selenoproteins and MASLD

#### 5.3.1 Selenoprotein P (SELENOP)

Previous research demonstrated that plasma Se concentration and two plasma selenoproteins, GPX3 and SELENOP, decreased in proportion to the severity of the cirrhosis of Child classes A, B, and C, as well as in control subjects (106). SELENOP, primarily produced in the liver, exhibited a similar decline. The study further reported that patients with cirrhosis do not exhibit Se deficiency, as evidenced by increased plasma GPx activity. To compare circulating SELENOP levels in patients with simple steatosis (SS) and MASH with those of healthy controls, a clinical study was conducted involving 31 patients with biopsy-proven MASLD (15 with SS, 10 with borderline MASH, and 6 with definite MASH) and 27 matched controls without MASLD (107). Definite MASH was associated with reduced SELENOP levels compared to controls, whereas SS and borderline MASH were not. This suggests that individuals with confirmed MASH exhibit lower SELENOP levels than controls, consistent with impaired hepatic SELENOP synthesis under conditions of cellular stress or inflammation.

In a separate case-control study involving 79 MASLD patients and 79 healthy controls, a positive correlation between MASLD and SELENOP was observed, with higher serum SELENOP levels as MASLD progressed (88). Further analysis revealed associations between SELENOP and MASLD risk factors, including BMI, alanine aminotransferase (ALT), gamma-glutamyl transferase (GGT), and serum uric acid. Additionally, SELENOP siRNA reduced triglyceride formation by activating AMPK/ACC, whereas SELENOP overexpression inhibited AMPK/ACC phosphorylation but exacerbated lipid accumulation. SELENOP serves as the primary transporter of Se to peripheral tissues and acts as a hepatokine with regulatory effects on insulin sensitivity and lipid metabolism. Evidence linking higher SELENOP concentrations to metabolic dysfunction and insulin resistance suggests that elevated levels may reflect impaired hepatic signaling pathways rather than improved Se availability.

Se homeostasis and SELENOP dynamics reveal a biphasic pattern during liver disease. In MASLD/MASH (early stages), SELENOP levels may increase (to support peripheral Se transport) and respond to IR-linked stress. However, during cirrhosis (advanced disease stage), hepatocellular damage reduces SELENOP production. This causes a decline despite oxidative stress and possibly an increase in GPx activity driven by non-transcriptional triggers. This contradiction may be attributed to variation in disease stage, hepatic synthetic capacity, and the dual function of SELENOP as a nutrient transporter and metabolic regulator.

#### 5.3.2 Selenoprotein M (SELENOM)

**Figure 3:**
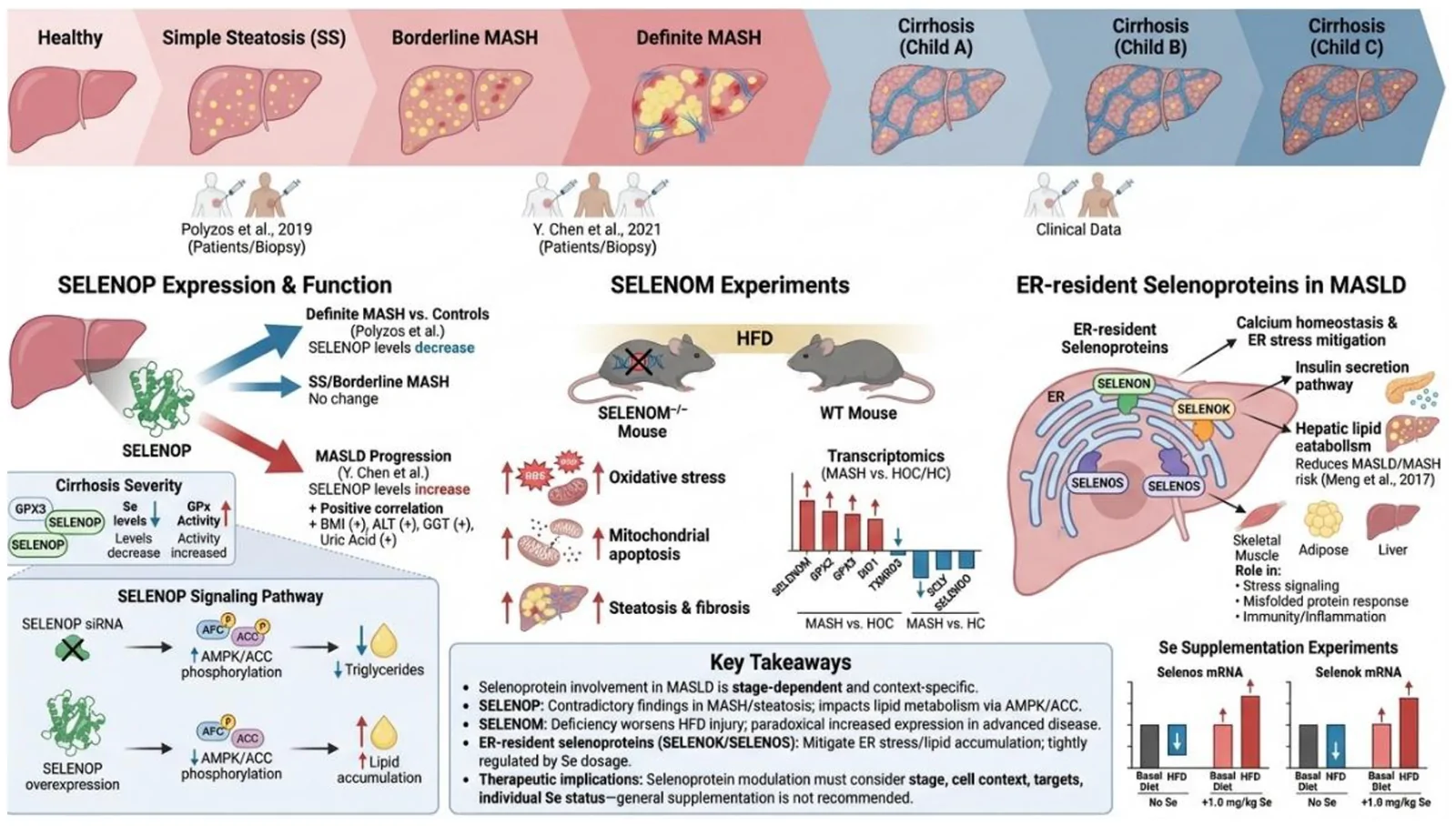
Mechanistic insights into the roles of specific selenoproteins in MASLD.

A recent study using SELENOM^−/−^ mice demonstrated that energy stress-induced downregulation of SELENOM in hepatocytes exacerbates high-fat diet (HFD)-mediated liver damage (108). SELENOM deficiency led to increased oxidative stress, mitochondrial apoptosis, inflammation, steatosis, and fibrosis in the livers of HFD-treated mice. This suggests that SELENOM is essential for maintaining mitochondrial quality control and mitigating lipotoxic liver damage. Therefore, SELENOM functions as an active regulatory node that connects energy stress, mitophagy, and the progression from steatosis to more advanced stages of liver disease. Another study assessed global gene expression in liver samples from patients with steatosis and MASH, and healthy controls, both with obesity (HOC) or without obesity (HC), to compare selenoprotein gene expression between healthy individuals and those with MASLD (109). TXNRD3 was reduced in both disease groups compared to HOC. SCLY (selenocysteine lyase) and SELENOO were lower in MASH than in HC. In contrast, SELENOM and other selenoproteins, including DIO1, GPX2, and GPX3, showed higher expression in MASH than in HOC (Figure 3). This pattern suggests compensatory upregulation of antioxidant and stress-response selenoproteins as liver injury progresses.

SELENOM provides cellular protection, but its expression appears to increase in advanced human disease, likely reflecting an insufficient adaptive response to ongoing oxidative and mitochondrial stress. These findings suggest that MASLD progression is characterized by coordinated, maladaptive changes in multiple selenoproteins, which may serve as mechanistic targets and biomarkers beyond their role as indicators of Se status. Table 3 summarizes the association of selenoprotein knockout/overexpression with MASLD progression in experimental animal models.

**Table 3:** Summary of experimental studies (animal model) on the association of Selenoprotein knockout/overexpression with MASLD progression.

References	Experimental animal model	Selenoprotein (knockout or overexpression)	Effects associated with selenoprotein knockout or expression	Key findings
Cai et al. (108)	C57BL/6J mice; male; 4-week-old	*Selenom*^+/+^ and *Selenom*^−/−^ mice were fed HFD and regular diets for 18 weeks.	Histopathology *Selenom*^−/−^: ↑ liver fibrosis ↑ hepatic vacuolization and steatosis, ↑ nuclear atrophy, and ↑ liver degeneration Biochemical markers *Selenom*^−/−^: ↑ ALT (47%) ↑ AST (33%) ↑ TG (18%) ↑ TC (29%) Molecular analyses *Selenom*^−/−^: ↑ Fibrosis-related genes (3–5-fold): *Cox I, Cox IV, Tgfb1, Timp1*, *Ccn1, Ccn2, Mmp9*	*Selenom*^−/−^ aggravated HFD-mediated hepatic steatosis, inflammation, and fibrosis
Liu et al. (195)	C57BL/6N mice; male; 8 weeks OLD	Forty each of *Txnrd*3^+/+^ and *Txnrd*3^−/−^ mice were randomly grouped into *Txnrd*3^+/+^, NC-*Txnrd*3^−/−^, TAA-*Txnrd*3^+/+^, and TAA-*Txnrd*3^−/−^ groups; mice were fed Thioacetamide (TAA) to induce liver cirrhosis.	Histopathology *Txnrd*3^−/−^: Large-area hepatocyte swelling Extruded hepatic sinusoids Hepatocytes with granular degeneration and granular cytoplasm Massive bile duct hyperplasia Connective tissue hyperplasia Several false vesicles Substantial stellate cell activation Scattered lymphocyte infiltration TAA-*Txnrd*3^−/−^: ↓ GPX4 (49 %) TAA-*Txnrd*3^+/+^: ↓ GPX4 (32 %) Biochemical markers TAA-*Txnrd3^−/−^*: ↑ ALT (116%) ↑ ALP (118%) ↓ GSH (43%)	More pronounced liver damage in *Txnrd*3^−/−^ mice
Qiao et al. (196)	C57BL/6 mice; male; 4-week-old	Mice (*Selenos^*f*/*F*^* and *Selenos^*H*/*KO*^*) were randomly divided into 2 groups: fed regular chow or HFD for 20 weeks.	Histopathology *Selenos^*H*/*KO*^*: Aggravated hepatosteatosis Dyslipidemia. Biochemical markers *Selenos^*H*/*KO*^*: ↑ ALT (33%) ↑ AST (17%) ↑ TG (20%) ↑ TC (23%) ↑ LDL-C ↑ FFA (7%) Molecular analyses *Selenos^*H*/*KO*^*: ↑*Cd36* (2.25-fold) ↑*Fatp2* (1.14-fold, ↑*Fatp5* (1.53-fold) ↓*Pparα* (0.6-fold) ↓*Cpt2* (0.5-fold) ↓*Acox1* (0.5-fold) *ER stress markers* ↑ GRP78 (2.46-fold) ↑ CHOP (2-fold) ↑ eIF2α (1.6-fold) ↑ JNK (1.3-fold) ↓ FGF21 (0.6-fold) ↓ *Fgf21*(0.5-fold)	*Selenos* overexpression inhibited hepatic steatosis and insulin resistance.
Zheng et al. (90)	C57BL/6 mice; male; 6–8 weeks old	WT and *Selenof*^−/−^ mice were randomly divided into two groups: Normal chow and HFD. Mice were fed for 24 weeks.	Histopathology *Selenof*^−/−:^ Severe hepatic steatosis Large lipid vesicles Islet hyperplasia Biochemical markers *Selenof*^−/−^: ↑ TC 45% ↑ LDL-c (33%) Molecular analyses ↑ GRP78 ↑ Calnexin ↓ Lipoprotein lipase (0.2-fold) ↓ Carboxylesterase 1D (0.3-fold)	*Selenof*^−/−^ contributes to glucose and lipid metabolism disorders
You et al. (197)	C57BL/6J mice; male; 8-week-old	Two-phase experiment HFD or normal chow diet group; fed for 16 weeks; *Selenok* full-length (WT) or SH3 binding domain truncated *Selenok* (SH3 BD TRUNC) were injected into C57BL/6J wild-type mice via the tail vein; Mice were maintained on an HFD for 8 weeks.	HFD:↑*Selenok (*1.3- 3-fold)^†^ ↑ ER distribution of SELENOK ↑ CD36 (3-fold) Truncated *Selenok*:↓ Hepatic steatosis ↓ Lipid accumulation ↓ Intrahepatic TG (71%) ↓ FFA (75%)	*Selenok* overexpression promotes hepatic lipid buildup and enhances CD36 subcellular trafficking.
Seale et al. (198)	C57BL/6J mice; male/female; *Scly^+/+^* and *Scly^−/−^*	*Scly^+/+^* and *Scly^−/−^* mice were fed standard lab chow containing 0.25 to 0.3 ppm of Se for 2 months.	Histopathology *Scly^−/−^*: Larger epididymal and inguinal WAT depots Impaired systemic glucose tolerance Hepatic steatosis (2x TG level in the liver) Biochemical markers *Scly^−/−^*: ↓ Liver Se (10%) Molecular analyses *Scly^−/−^*: ↑*GPx1* (7-fold) ↑*GPx4* (5-fold) ↑*Selenoi* (4-fold) ↑*Selenok* (1.4-fold) ↑*Selenor* (2-fold) ↑*Selenos* (2-fold) ↑*Selenot* (3-fold) ↑*Selenow* (2-fold) ↑*Sep15* (5-fold) ↑*Selenop* (5-fold) ↑*Txnrd1* (3-fold) ↑*Txnrd2* (3-fold) ↑*Txnrd3* (2-fold)	*Scly^−/−^* increases hepatic lipogenesis and attenuates insulin signaling, exacerbated by a low-Se diet.

^†^Protein and mRNA level.

MMP1, matrix metalloproteinase 1; SELENOM, selenoprotein M; AST, aspartate aminotransferase; ALT, alanine aminotransferase; HFD, high-fat diet; COX1, cytochrome c oxidase subunit 1; COX IV, cytochrome c oxidase subunit IV; CCN1, cellular communication network factor 1; TGFB1, transforming growth factor beta 1; TIMP1, tissue inhibitor of metalloproteinases 1; CCN2, cellular communication network factor 2; MMP9, matrix metalloproteinase 9; TXNRD, thioredoxin reductase; ALP, alkaline phosphatase; GSH, glutathione (reduced); GPX4, glutathione peroxidase 4; SELENOS, selenoprotein S; FFA, free fatty acids; LDL-C, low-density lipoprotein cholesterol; HDL-C, high-density lipoprotein cholesterol; FATP5, fatty acid transport protein 5; PPARα, peroxisome proliferator-activated receptor alpha; CPT2, carnitine palmitoyltransferase 2; ACOX1, acyl-CoA oxidase 1; GRP78, glucose-regulated protein 78; CHOP, C/EBP homologous protein; eIF2α, eukaryotic translation initiation factor 2 alpha; JNK, c-Jun N-terminal kinase; FGF21, fibroblast growth factor 21; SCLY, selenocysteine lyase; WAT, white adipose tissue.

#### 5.3.3 SELENON, SELENOT, SELENOS, and SELENOK

Four ER-selenoproteins, SELENON, SELENOT, SELENOS, and SELENOK, are considered essential for regulating intracellular calcium concentration and mitigating ER impairment, potentially influencing the pathological processes of HFD-induced MASH (110). SELENOK has been linked to increased insulin secretion in the ER and may reduce the incidence of MASLD and MASH by promoting hepatic lipid catabolism (111). SELENOS, a transmembrane protein widely distributed in tissues such as skeletal muscle, adipose tissue, and liver, is involved in stress signaling. This includes inhibiting diverse adverse effects of misfolded proteins, which are linked to immunity and inflammation (112). Additional studies indicate that in both basal-diet- and high-fat diet-fed mice, the highest *Selenos* mRNA levels were observed in groups supplemented with 1.0 mg/kg Se. In contrast, the highest *Selenok* expression was detected only in the 1.0 mg/kg Se basal diet groups (110). These findings reinforce the view that MASLD is characterized not only by altered lipid flux but also by impaired resilience to organelle stress and signaling crosstalk, particularly in the ER. This provides a mechanistic rationale for examining selenoproteins, such as SELENOK and SELENOS, as biomarkers for MASLD and MASH.

#### 5.3.4 SELENOH

A recent study identified the Se-SELENOH-PPARα axis as a critical pathway linking Se homeostasis to MASH development (23). Reduced hepatic Se status, impaired Sec incorporation, and decreased translation of multiple stress-responsive selenoproteins, including GPX1, MSRB1, and SELENOH, are associated with increased severity of liver injury and fibrosis. SELENOH was shown to localize predominantly to the nucleus, bind ligand-activated PPAR, recruit the coactivator P300, and stimulate transcription of fatty acid oxidation genes. Loss of *Selenoh* exacerbated MASH phenotypes and suppressed FAO activity. In contrast, SELENOH overexpression had the opposite effect, functioning as a nuclear PPAR coactivator to promote fatty acid oxidation and protect against steatohepatitis. This suggests that SELENOH-dependent PPARα-mediated transcriptional control is essential for liver fatty acid oxidation.

## 6 Se and obesity

### 6.1 Se deficiency and obesity

A recent study involving 6,440 men and 6,849 women found that higher body mass index (BMI) was inversely associated with serum Se (Se) levels (113). The study also showed that individuals in the highest quartile of percent body fat (BF%) exhibited lower serum Se levels in both sexes. Similarly, women with morbid obesity demonstrated approximately 15% lower Se levels compared to lean controls (114). An analysis of 573 school children in Madrid reported that overweight children (BMI > P85) had serum Se levels and Se intake that were 14% and 27% lower, respectively, than those of children with normal weight (115). In metabolic syndrome, plasma Se levels were inversely associated with circulating MCP-1. In individuals with visceral obesity, plasma Se levels were strongly correlated with high-density lipoprotein cholesterol (HDL-C) and fatty acid-binding protein 4 (FABP4) (116). These findings support the hypothesis that obesity and metabolic syndrome are characterized by elevated oxidative and inflammatory stress, potentially resulting in relatively depleted or insufficient Se-dependent defenses.

Se treatment at concentrations near the physiological range (0, 5, 10, 20 ng/mL) significantly enhanced the differentiation of 3T3-L1 preadipocytes by 20% by modulating cell cycle signaling (G2/M progression) and upregulating cyclin-dependent kinases (CDK1 and CDK2) through the downregulation of CDK inhibitors (CDKNs) p21 and p27. Activation of the PI3K/Akt pathway was observed, whereas the MAPK1 (ERK2) pathway was not affected. Upregulation of matrix metallopeptidases 2 and 9 was associated with the Se-induced increase in preadipocyte migration (117). This indicates that within physiological conditions, Se modulates adipose tissue plasticity by promoting precursor cell proliferation, differentiation, and motility through the PI3K/Akt-CDK signaling pathways and MMP-mediated extracellular matrix remodeling.

### 6.2 Se supplementation and obesity

In an RCT involving 84 premenopausal women with central obesity, Se supplementation (200 μg/day) significantly reduced fasting serum insulin concentrations and the Homeostasis Model Assessment of IR Index. Coadministration with L-arginine (5 g/day) further decreased fasting NO concentrations (118). Recent research has demonstrated that facultative protein selenation, a novel non-canonical form of Se incorporation into proteins, occurs at elevated levels with SeMet supplementation. This process altered key metabolic proteins in mouse brown adipose tissue and conferred protection against obesity by increasing thermogenesis (119). This indicates that, within a defined dosing range, Se may improve insulin resistance and NO-mediated vascular function and reprogram brown adipose tissue to increase energy expenditure. These cases are context-specific (centrally obese premenopausal women; mouse models with high SeMet exposure) and support a mechanistic and translational hypothesis. However, they do not justify a general recommendation for high-dose Se supplementation.

Tinkov et al. (120) reported that sodium selenate (Na_2_SeO_4_) inhibits adipocyte development and induces cell death in 3T3-L1 cells at supraphysiological concentrations (100, 200, and 400 μM) by activating AMPK and phosphorylating acetyl-CoA carboxylase (ACC). Supraphysiological but non-cytotoxic concentrations of Na_2_SeO_4_ (1, 10, 25, 50 μM) decrease the expression of peroxisome proliferator-activated receptor gamma (PPARγ) and CCAAT-enhancer binding protein beta (C/EBPβ) without cytotoxic effects. At 50 μM, Na_2_SeO_4_ reduces adipocyte development by downregulating PPARγ, C/EBPα, and leptin gene expression (121). Selenate, but not selenite or methylseleninic acid, administered at supraphysiological doses (5, 10, 25, 50 μM), inhibits adipogenic differentiation of preadipocytes through activation of transforming growth factor-β1 (TGF-β1) and disruption of C/EBP signaling, without affecting cell viability (122). Selenate (50 μM) exerts an antiadipogenic effect that is evident only during the early stages (0–2 days) of adipogenesis in 3T3-L1 cells, consistent with reduced adipogenesis at high selenate levels without cytotoxicity (121). This highlights selenate's dose-dependent, *in vitro* anti-adipogenic effects, mediated by energy sensing via AMPK activation and transcriptional repression. The influence of Se on adipocyte development is shaped not only by its chemical speciation but also by the timing of exposure, both of which serve as key determinants of its biological effects.

Animal studies reveal that a supraphysiological dose of Na_2_SeO_4_ (0.72 mg/kg/day for 8 weeks) administered to high-fat diet (HFD)-fed mice significantly reduces adiposity by downregulating PPARγ signaling. This process is accompanied by decreased expression of diacylglycerol O-acyltransferase 2 (DGAT2) and leptin, as well as improved insulin sensitivity (123). Conversely, in HFD-fed rats, Na_2_SeO_3_ treatment (0.3 mg/kg diet for 4 weeks) decreases the expression of genes involved in fatty acid synthesis, including fatty acid synthase (FAS), lipoprotein lipase (LPL), PPARγ, and sterol regulatory element-binding protein 1 (SREBP1), while increasing the expression of genes associated with lipid catabolism (124).

In HFD-fed mice, pretreatment with supraphysiological Se levels (29.13 mg/L in drinking water for 2–4 months) increased adipocyte differentiation. Ectopic lipid accumulation was reduced by modulating PPARγ and C/EBPα/β, whereas Se posttreatment enhances lipolysis and subsequent ectopic lipid accumulation via the protein kinase A (PKA)/hormone-sensitive lipase (HSL) pathway (125). Administration of SeMet at 150 μg/kg to HFD-induced obese mice conferred significant protection against diet-induced obesity, IR, and hepatic and renal lipid accumulation. This is characterized by enhanced beiging of white adipose tissue and increased energy expenditure (126). The length and amount of Se exposure have a two-phase, time-dependent effect that strongly influences adipogenesis. Prolonged pretreatment promotes adipocyte differentiation and reduces ectopic lipid accumulation, whereas shorter pretreatment periods or posttreatment promote lipolysis and reduce fat accumulation. This is evidence that the role of Se in body fat and insulin sensitivity may depend on dose (physiological or supraphysiological), chemical form (selenate or selenite), and, especially, the timing and duration of exposure relative to HFD-induced obesity.

### 6.3 Selenoproteins and obesity

#### 6.3.1 SELENOP

The SELENOP gene is expressed in rat adipose tissue, suggesting a role in adipocyte physiology (127). Lower SELENOP levels are associated with impaired adipogenesis, insufficient antioxidant selenoproteins, oxidative stress, and preadipocyte inflammation. Adipocyte differentiation is inhibited when normal 3T3-L1 cells are co-cultured with *Selenop*^−/−^cells (128). SELENOP deficiency prevented diet-related increases in adipose tissue formation and adipocyte hypertrophy, despite higher calorie intake in the *Selenop*^−/−^ mice in comparison to wild-type mice (129). *Selenop* gene overexpression in C/EBPα-NIH3T3 cells promotes adipogenic differentiation (130).

Mature adipocytes exposed to omentin *in vivo* exhibit a reduction in SELENOP levels, along with increased inflammation (131). *Selenop* is also reduced in 3T3-L1 adipocytes following treatment with TNF-α or H_2_O_2_. This suggests a link between oxidative stress, adipose tissue inflammation, and changes in selenoprotein activity when obesity is expressed phenotypically (132). Low oxygen levels increased proinflammatory cytokine levels (IL6 and MCP1) in preadipocytes, and these increases were correlated with lower SELENOP levels (133). Insulin-resistant individuals, *ob/ob* mice, HFD-induced obese mice, and Zucker rats exhibit reduced *Selenop* gene expression in adipose tissue (132). In contrast, leptin treatment in *ob/ob* mice shifted gene expression to increase fat breakdown by blocking SREBP1 signaling and elevating *Selenop* and SREBP1 expression in the liver (134). However, adipose tissues from obese OLETF rats exhibited a twofold increase in *Selenop* expression (127).

Results from these studies indicate that SELENOP acts as a redox-sensitive regulator of adipocyte differentiation and fat metabolism. SELENOP maintains adipose tissue homeostasis under normal conditions, but not when obesity or insulin resistance is phenotypically expressed. Therefore, SELENOP may serve as a biomarker and treatment target linking Se metabolism with fat tissue inflammation and energy balance.

#### 6.3.2 SELENOS

Dexamethasone-induced proteasomal degradation of SELENOS promotes adipogenesis during the early stages of cell differentiation. At the same time, SELENOS overexpression reduces ER stress, ER stress-dependent adipogenesis, C/EBP levels, and other adipocyte secretory products (135). *Selenos* knockdown was shown to increase apoptotic cell death via the IRE1α-sXBP1-p-JNK pathways and ERS (136). The suppression of adipogenesis by non-cytotoxic selenate in 3T3-L1 preadipocytes may also result from alterations in SELENOS-mediated ERS. In obese individuals, SELENOS expression in adipose tissue was higher (120).

SELENOS gene expression levels were correlated with body measurements that are indicative of obesity [BMI: *r* = 0.25 (*p* ≤ 0.05), sagittal diameter: *r* = 0.31 (*p* ≤ 0.01), HDL-c: *r* = −0.35 (*p* ≤ 0.01), triglycerides: *r* = 0.27 (*p* ≤ 0.05), insulin: *r* = 0.30 (*p* ≤ 0.01), and HOMA-IR: *r* = 0.30 (*p* ≤ 0.05)] (137). Insulin increases SELENOS levels in human adipocytes *in vitro*, suggesting a connection between IR and SELENOS expression in obesity (137). Moreover, *Selenom*^−/–^ may influence how the brain regulates energy expenditure, as it is associated with greater weight gain and increased adipose tissue growth, partly due to hypothalamic leptin resistance (138). This suggests that SELENOS plays dual roles: it suppresses early-stage adipogenesis by reducing ER stress and C/EBP levels. However, it is upregulated in obese people and correlates with anthropometric measures of obesity and IR, suggesting compensatory responses.

#### 6.3.3 GPX1

Changes in GPX protein levels and activity contribute to the development of obesity and related metabolic disorders. GPX1 overexpression in mice fed a standard diet resulted in elevated leptin levels, increased adiposity, increased body weight, and impaired insulin sensitivity. GPX1 overexpression was also associated with a large decrease in insulin-induced phosphorylation of the insulin receptor β-subunit in the liver and of Akt in both liver and muscle, suggesting a disruption in the redox regulation of insulin signaling (139). Furthermore, the incidence of hepatic steatosis and IR was substantially lower in *Gpx*1^−/−^ mice fed a HFD (140).

Gpx1-deficient epididymal adipocytes exhibited reduced cell diameter while maintaining normal differentiation status, as indicated by PPARγ, AP2, and C/EBP expression. This is further evidence that *Gpx*1^−/−^ mice are generally resistant to diet-induced obesity (141). Similarly, a high-fat and sucrose diet led to a deteriorated metabolic profile, including poor glucose control, excessive fat accumulation, and cardiac fibrosis in mice with GPX haploinsufficiency (*Gpx1*^+/−^) (142). The adverse effects of GPX1 overexpression on lipid and glucose metabolism in male mice were alleviated by dietary Se deficiency and/or food restriction (143). In *Gpx*1^−/–^ mice, there was partial improvement in insulin secretion in the presence of glucose (144).

The effects of Se status and energy restriction on adverse metabolic outcomes, including increased adiposity, insulin resistance, and hepatic lipid accumulation, demonstrate the sensitivity of GPX1'swith respect to regulating oxidation. Maintaining an optimal balance of GPX1 activity, rather than solely enhancing its antioxidant capacity, is essential for preventing metabolic problems associated with obesity.

#### 6.3.4 DIO2

*Dio*2^−/−^ mice had increased susceptibility to obesity when fed an HFD (145). *Dio*2^−/−^ mice exhibited a higher risk of obesity at thermoneutral conditions (30 °C) than at normal room temperature (22 °C) (146). In contrast, neonatal hepatocyte-specific *Dio*2^−/−^ mice were less likely to develop obesity and fatty liver (147). Furthermore, DIO may contribute to obesity by regulating heat production in brown adipose tissue (BAT) (148). While DIO may serve as a key metabolic regulator, its effects on obesity and fat metabolism differ across tissue types, developmental stages, and environmental factors, such as temperature.

#### 6.3.5 SELENOV

In C57BL/6J mice, additional evidence for the protective role of *Selenov*^−/−^ in diet-induced obesity is provided by downregulation of genes involved in lipid catabolism and upregulation of genes associated with lipid anabolism (149). This occurs alongside increased body weight and adipose tissue accumulation, as well as higher levels of *Fasn, Acaca, Dgat1*, and *Lpl* genes in white adipose tissue (WAT).

Overall, the impact of selenoproteins on obesity is context-dependent (Table 4). Each family member exerts variable effects depending on tissue type (white and brown/beige fat, and visceral versus subcutaneous fat) and expression levels. These differences collectively influence adipogenesis, inflammation, and metabolic function. However, many studies measure only overall Se or plasma GPx as indirect indicators, limiting mechanistic interpretation of epidemiological associations. These results suggest that broad Se supplementation is not ideal and support the development of treatments targeting specific selenoproteins based on individual metabolism and tissue needs. Furthermore, mouse models indicate that Se supplementation can protect against diet-induced obesity and mimic some hallmarks of methionine restriction. However, these results vary widely depending on mouse strain, age, and dietary background, and so far, there is no clear equivalent to human obesity treatments.

**Table 4:** Summary of tissue-specific effects of various selenoproteins in different metabolic diseases.

Selenoprotein	Main tissue (s)/cell type (s)	MASLD	Obesity	CVD	Main interpretation
SELENOP	Liver, adipose tissue	Circulating levels change with disease stage; lower in definite MASH in one study, higher with MASLD severity in another. Promotes lipid accumulation when overexpressed; loss reduces triglyceride formation via AMPK/ACC.	Lower adipose SELENOP is linked to impaired adipogenesis, oxidative stress, and inflammation. Deficiency limits adipose expansion; overexpression promotes adipogenesis.	Low circulating SELENOP is associated with metabolic syndrome and mortality. Knockout reduces ischemia/reperfusion injury and infarct size.	Reveals a biphasic pattern during liver disease; regulator of lipid metabolism, insulin sensitivity, and redox balance
SELENOM	Liver (mitochondria/ER)	Protects against HFD-induced liver injury. Deficiency increases oxidative stress, apoptosis, steatosis, and fibrosis; higher in MASH vs controls in human samples.	No strong direct obesity phenotype highlighted but linked to stress responses relevant to obesity.	–	Likely protective in liver stress and disease progression
SELENON	Liver (ER)	Included among ER proteins involved in calcium homeostasis and MASH pathogenesis	No specific obesity phenotype reported	–	ER stress and calcium regulator with likely indirect metabolic relevance
SELENOT	Liver (ER)	Grouped with ER selenoproteins that preserve ER function in MASH	No specific obesity phenotype reported	–	Stress-response selenoprotein with possible metabolic relevance
SELENOS	Adipose tissue, liver, skeletal muscle, endothelium	Involved in ER stress control and MASH-related stress signaling; higher hepatic expression noted in MASH	Suppresses early adipogenesis by reducing ER stress. In obesity, adipose expression is higher and correlates with BMI, insulin, sagittal diameter, HOMA-IR, and triglycerides	Reduces endothelial inflammation, ROS, endothelin-1, and monocyte adhesion; increases NO-related signaling	Protective against ER stress and vascular inflammation, but may rise as a compensatory response in obesity
SELENOK	Liver (ER), macrophages	May support hepatic lipid catabolism and reduce MASLD/MASH risk	Limited direct obesity data, but may improve insulin secretion and lipid handling	Present in aortic plaques; knockout reduces atherosclerosis and lesion formation	Linked to ER homeostasis and macrophage-driven atherogenesis
GPX1	Liver, muscle, adipose tissue, endothelium	Overexpression worsens insulin resistance and fat accumulation; knockout protects against steatosis and metabolic dysfunction.	Overexpression increases body weight, adiposity, leptin, and insulin resistance; deficiency protects against diet-induced obesity.	Preserves endothelial NO and limits inflammatory signaling; reduces vascular dysfunction	Beneficial in vascular tissue, but excess GPX1 may worsen metabolic disease
GPX2/GPX3	Liver	Increased in MASH and cirrhosis-related liver stress	–	–	Stress-responsive antioxidant markers in progressive liver disease
DIO2	Liver, BAT, heart	Hepatic loss alters susceptibility to fatty liver in a stage-dependent manner.	Knockout increases obesity risk; role linked to thermogenesis and energy expenditure.	Overexpression improves cardiac contraction and SERCA2a in pressure overload.	Strong tissue-specific effects on energy balance and cardiac function
TXNRDs	Cardiomyocytes	–	–	Low Se reduces TXNRDs in cardiomyocytes.	Important for cardiac redox control
SELENOV	WAT	–	Deficiency promotes weight gain, adipose expansion, and lipogenesis.	–	Supports lipid catabolism and protection against obesity
SELENOH	Liver (nucleus)	Promotes PPARα-driven fatty acid oxidation; loss worsens MASH and suppresses FAO.	–	–	Nuclear coactivator that protects against steatohepatitis

SELENOP, selenoprotein P; MASH, metabolic steatohepatitis; MASLD, metabolic dysfunction–associated steatotic liver disease; AMPK/ACC, AMP-activated protein kinase/acetyl-CoA carboxylase; SELENOP, selenoprotein P; HFD, high-fat diet; ER, endoplasmic reticulum; ROS, reactive oxygen species; NO, nitric oxide; HOMA-IR, homeostatic model assessment of insulin resistance; WAT, white adipose tissue; BAT, brown adipose tissue; GPX1, glutathione peroxidase 1; TXNRD, thioredoxin reductase; PPARα, peroxisome proliferator–activated receptor alpha; FAO, fatty acid oxidation; SERCA2a, sarcoplasmic/endoplasmic reticulum Ca^2+^-ATPase 2a; GPX2, glutathione peroxidase 2; SELENOH, selenoprotein H; SELENOV, selenoprotein V; DIO2, iodothyronine deiodinase 2; SELENOT, selenoprotein T; SELENOS, selenoprotein S; SELENON, selenoprotein N; SELENOK, selenoprotein K; SELENOM, selenoprotein M.

While several human studies have linked Se levels to metabolic risk factors, including abdominal adiposity, blood pressure, dyslipidemia, and inflammation markers, these phenotypes frequently co-occur with obesity and CVD, complicating efforts to isolate adiposity as the primary endpoint. Additionally, obesity independently disrupts redox homeostasis, induces endoplasmic reticulum stress, and impairs liver function, all of which can influence selenoprotein synthesis and circulating Se levels. Therefore, lower SELENOP or GPx activity in individuals with obesity might reflect adaptive responses or increased Se utilization in response to oxidative stress, rather than a true deficiency. This complexity complicates interpreting “low levels” as a simple indicator of Se exposure.

## 7 Se and CVD

### 7.1 Se deficiency and CVD

Se deficiency (< 0.025 mg/kg) in adult C57/BL6 mice contributed to reactive myocardial fibrosis, reduced systolic function, decreased *Gpx1* expression and activity, and increased oxidative stress (150). In a clinical study comparing Se levels in 20 patients with congestive heart failure to those of healthy people, only 6 patients had levels similar to those of the healthy group. Fourteen had much lower levels (mean 47.8 ± 16.2 g/L), which correlated with left ventricular ejection fraction, an indicator of cardiac contractility (151). Se intake and blood levels were lower in heart failure patients (152). However, left ventricular ejection fraction was not associated with Se levels, although it was related to peak oxygen consumption. Inadequate Se intake or status may be a risk factor for developing or worsening heart failure caused by oxidative stress and myocardial damage and fibrosis. Still, how much Se levels directly affect left ventricular ejection fraction in all heart failure patients is unclear. Se status seems more closely linked to overall function and outlook than to a single measure of systolic function.

### 7.2 Se supplementation and CVD

Prolonged Se supplementation (200 μg/day) for 8 weeks reduced inflammation, improved insulin sensitivity, and increased plasma total antioxidant capacity in patients with T2D and coronary heart disease (CAD) (153). Se may also reduce NF-κB activity, thereby lowering inflammatory responses (154). However, a single intravenous dose of 600 μg Se administered before heart surgery did not change IL-6, TNF-α, or CRP levels (155). In another study, supplementation with Se (200 μg organic Se) and coenzyme Q10 (200 mg/day) in patients with CVD led to a lower inflammatory status and much lower cardiovascular mortality after 10 years. However, differences in dosage, supplement form, and duration prevent the direct comparison between studies (156). Also, because Se was given with coenzyme Q10, the observed effects cannot be solely attributed to Se.

Se supplementation at 2 μg/kg/day for 6 months, along with anti-failure therapy, was given to a 15-month-old patient with Se-responsive cardiomyopathy, poor growth, long-lasting fever, and breathing problems (157). This treatment improved cardiac function, hair and skin health, and overall health. In another study, 145 individuals with CAD, with or without metabolic syndrome, received daily Se supplementation (200 mg) for two months (158). Plasma Se, SELENOP, and *Selenop* mRNA levels did not change during this time. After 60 days of Se supplementation in 41 CAD patients, levels of matrix metalloproteinase 9 and cyclooxygenase-2, key enzymes involved in atherosclerosis, remained unchanged (159). Thus, in populations without clear Se deficiency, supplementation may not significantly alter Se status or enzymes associated with arterial disease. Moreover, the duration and dosage of supplementation in these studies might not have been sufficient to lead to noticeable changes.

Treatment with Na_2_SeO_3_ conferred resistance to 20-HETE-induced endothelial barrier loss, while Vitamin E alone did not prevent complete barrier disruption (160). Dimitrov et al. (161) reported that Se supplementation (Na_2_SeO_3_; 2 ppm) maintained heart tissue structure in rabbits with Adriamycin-induced cardiomyopathy. Collectively, these results suggest that Se can protect against oxidative and structural damage in endothelial cells and in animal models of heart disease, likely by acting as an antioxidant and thereby preserving tissue structure.

In rats, 8 weeks of Se supplementation (2.5 mg/kg) increased cardiac mitochondrial GPx activity, lowered malondialdehyde levels in rats treated with Adriamycin, and reversed reperfusion-induced structural alterations in their hearts (162). However, mice fed a Se-supplemented diet (0.5 mg/kg) for 12 weeks developed compromised myocardial matrix gene expression. This resulted in reactive myocardial fibrosis and diastolic dysfunction without cardiac hypertrophy, ultimately resulting in altered myocardial matrix remodeling and heart failure (150). In hypertensive rats, supplementation with selenomethionine (SeMet; 0.25 μg Se/g) increased GPX-1 activity in the blood and aorta. Concurrently, eNOS-3 levels and aortic wall lipid peroxidation declined, as did blood levels of antibodies against advanced glycation end products (163). Different supplementation methods and results complicate cross-study comparison. Moreover, some Se treatments caused harmful effects (e.g., myocardial fibrosis in mice), indicating risk with over-supplementation of Se.

These data generally suggest a link between low Se levels and higher CVD occurrence. However, some studies show that Se can lower inflammation, improve insulin sensitivity, boost antioxidant levels, and protect heart function, especially when Se is low or in cardiotoxic conditions.

### 7.3 Selenoproteins and CVD

#### 7.3.1 DIO2

In a pressure-overload hypertrophy model, DIO2 overexpression in mice improved heart muscle contraction. This was associated with higher SERCA2a levels, due to increased Ca2+ uptake into the sarcoplasmic reticulum (164). Higher DIO2 activity affects SERCA levels because deiodinases regulate thyroid hormone levels, which, in turn, modulate SERCA levels in both skeletal and heart muscles (165). This suggests that DIO2 improves heart muscle contraction.

#### 7.3.2 TXNRDs

Quantitative RT-PCR analysis showed that several selenoproteins, including all three TXNRDs, were significantly reduced after the administration of a low Se (0.033 mg/kg) to chicken cardiomyocytes and thioredoxin knockdown via siRNA (166). However, it remains unclear whether this change is adaptive or maladaptive (167). These findings suggest that thioredoxin levels in cardiomyocytes may regulate TXNRD expression.

#### 7.3.3 SELENOP

*Selenop*^−/−^ mice subjected to ischemia/reperfusion (I/R) injury exhibited significantly smaller infarct sizes, showing less cardiac apoptosis and tissue injury, along with increased phosphorylation of multiple Akt, Erk, and other RISK pathway proteins when compared to wild-type controls (168). Decreased circulating SELENOP levels have been associated with a higher risk of metabolic syndrome, as well as increased mortality in acute heart failure and all-cause cardiovascular mortality (169–171). Hence, SELENOP may worsen ischemia/reperfusion (I/R) injury in the short term but help protect the heart and metabolic health and support survival in the long term.

#### 7.3.4 GPX1

In primary aortic endothelial cells from *Gpx*^−/−^ mice, treatment with TNF-α reduced Akt activation and NO bioavailability, while prolonging IκB degradation and increasing the activation of p38, ERK, and JNK (172). These changes indicate increased NF-κB activity and vascular inflammation. Similarly, increased *VCAM-1* expression was observed in cell lines and aortas (173). GPX-1 administration attenuated these effects, highlighting the importance of GPX-1 antioxidant defense in controlling vascular inflammation and endothelial dysfunction in CVD.

#### 7.3.5 SELENOK and SELENOS

SELENOK expression has been detected in aortic plaques, especially within macrophages. Knockout of SELENOK in mice resulted in less atherosclerosis, as evidenced by fewer lesion formations, suggesting a potential role in foam cell generation and atherogenesis (174). SELENOS overexpression mitigated the adverse effects of TNF-α treatment on human umbilical vein endothelial cells (HUVECs) by lowering ICAM-1 and VCAM-1 expression, inhibiting THP-1 monocyte adhesion, and reducing inflammation-related gene expression (including IL-1, IL-6, IL-8, and MCP-1), despite activation of MAPK and NF-κB pathways. SELENOS also lowered TNF-α-induced increases in ROS and endothelin-1 levels, while increasing eNOS and NO levels (175).

In human aortic endothelial cells (HAECs), SELENOS overexpression decreased the p-PKCα/PKCα ratio, ROS, and endothelin-1 levels, while increasing the p-eNOS/NOS and p-Akt/Akt ratios, total PI3K, SOD1, and SOD2, indicating indirect activation of the PI3K/Akt/eNOS signaling pathway (176, 177). SELENOS knockdown reversed these effects on endothelial cells, indicating that SELENOS protects against endothelial injury caused by high glucose and oxidized LDL. Hence, SELENOS could be a potential target for preventing and treating diabetic vascular complications by regulating HAEC's autophagy through activation of the Akt/mTOR signaling pathway.

Selenoproteins are implicated in CVD at multiple stages, including endothelial dysfunction, oxidative modification of lipoproteins, plaque stability, myocardial ischemia-reperfusion injury, and heart failure remodeling. However, many studies use general outcomes. The absence of stratification by disease stage or phenotype, such as heart failure with preserved vs. reduced ejection fraction, ischemic vs. non-ischemic cardiomyopathy, or stable vs. acute coronary syndrome, may weaken the specific role of Se.

## 8 Perspectives

The association between Se and metabolic disease appears to vary depending on disease stage, Se exposure, metabolic context, and the biomarker assessed. Accumulating evidence indicates that Se status and the broader selenoprotein network, including GPX1, SELENOP, SELENOM, SELENOS, SELENOK, DIO2, and SELENOH, contribute to metabolic disease progression through distinct but complementary, tissue-specific mechanisms. Nevertheless, current findings are inconsistent, particularly when comparing animal and human studies. The therapeutic value of Se supplementation, both preventive and interventional, remains to be defined through RCTs that assess efficacy, safety, and dosing in patients at different disease stages. Future studies should establish optimal Se levels for liver health and for the progression of metabolic disease and identify the groups most at risk.

Determining the causal relationship between Se deficiency and liver disease progression requires large-scale longitudinal studies across diverse populations. Mechanistic studies employing molecular and genetic approaches are needed to elucidate how Se influences hepatic antioxidant and inflammatory pathways, including their molecular targets in oxidative stress and immune regulation. Future research should combine mechanistic experiments with rigorous longitudinal human studies to establish causality and evaluate whether selenoproteins may serve as clinically relevant biomarkers or therapeutic targets in different disease states.

Current research on Se and selenoproteins in metabolic diseases predominantly employs conventional animal models, such as those with HFD-induced obesity, while largely neglecting the influence of aging. This is due to paucity of data from aging-specific models. Consequently, the specific contributions of Se to age-related metabolic disorders remain insufficiently characterized. Age-dependent variations in redox status, inflammatory tone, and insulin sensitivity may influence the metabolic effects of Se and selenoprotein activity. Age-stratified analyses are needed to clarify how Se and selenoprotein activity affect metabolic disease across the lifespan.

Moreover, the effect of Se on metabolic regulation may depend on the composition of the gut microbiota, acting through the gut-liver and gut-adipose tissue axes. Variation in selenoprotein expression across metabolic tissues, particularly among adipose subtypes, may also lead to tissue-specific responses to Se, limiting the generalizability of findings from metabolic disease. Hence, future studies could incorporate diverse metabolic models and explore interventions that account for heterogeneity to improve translational outcomes.

The considerable variability in dietary Se status, combined with its bimodal biological effects at low and high intake levels, underscores the need for precision nutrition strategies that prioritize insulin sensitivity as a central therapeutic target. These strategies must consider factors such as individual Se status, baseline IR, and metabolic phenotype to establish optimal supplementation thresholds that maximize insulin-sensitizing effects while minimizing risks in age-related chronic metabolic diseases. A predictive model can be developed to elucidate the conditions under which a specific selenoprotein is more likely to exert harmful or beneficial effects.

Recent advancements in omics datasets and analytical platforms, including SETRIP (for selenoproteome dynamics and turnover) (178) and heteroatom-tagged proteomics for absolute quantification of the plasma or serum selenoproteome (179), offer opportunities to construct mechanistic, data-driven predictive models relevant to Se biology. Such a model would integrate baseline Se status (plasma or serum Se, SELENOP, GPX activity), tissue or cell type (liver, muscle, adipose, immune cells), metabolic or inflammatory state (obesity, MASLD, insulin resistance), and genetic or epigenetic context (selenoprotein gene SNPs, SECIS variants, Sec machinery expression, methylation status), as well as exposure time and dose (acute versus chronic, dietary versus high-dose supplementation, Se species).

In conclusion, future research should prioritize 1) understanding how Se and selenoproteins modulate IR across different tissues and disease stages, 2) defining Se intake ranges that optimize insulin sensitivity in specific metabolic conditions, and 3) elucidating selenoprotein-specific pathways that contribute to the regulation of glucose and lipid metabolism. Translating these findings into clinical guidance requires systematic evidence grading and consensus on what constitutes optimal Se status, with attention to genetic and metabolic variations that may shape individual benefit. Until such frameworks are established, a precautionary approach is warranted: supplementation should be limited to cases of confirmed or likely deficiency, dietary sources should be prioritized, and the U-shaped risk of Se exposure should be clearly communicated to patients.
